# Treatment of an Intracranial Fusiform Vertebral Aneurysm Using the MVP® Micro Vascular Plug System as an Adjunct to Pipeline(TM) Embolization: Technical Case Instruction

**DOI:** 10.7759/cureus.57062

**Published:** 2024-03-27

**Authors:** Christian Ferreira, Ramesh Grandhi, Márcio Yuri Ferreira, Richard Williamson, Ricardo Hanel

**Affiliations:** 1 Department of Neurosurgery, Lenox Hill Hospital, New York, USA; 2 Neurosurgery, Utah University, Salt Lake City, USA; 3 Neurosurgery, Ninth July University, New York, USA; 4 Neurosurgery, Allegheny Health Network, Pittsburgh, USA; 5 Neurosurgery, Baptist Medical Center Jacksonville, Jacksonville, USA

**Keywords:** mvp® microvascular plug, pipeline embolization device, fusiform aneurysms, endovascular treatment, case report

## Abstract

Fusiform and dolichoectatic aneurysms pose unique challenges in treatment, often requiring alternative approaches compared to saccular aneurysms. Microsurgical options like clipping or a bypass can be difficult, leading to the advancement of endovascular techniques. Flow-diverting stents and vessel sacrifice with detachable coils have shown efficacy in reconstruction. The MVP® Micro Vascular Plug System (Medtronic, Minneapolis, Minnesota, USA) offers a resheathable plug for vessel occlusion through electrolytic detachment. This case report illustrates the supplementary application of MVP® subsequent to flow diverter (FD) stenting, resulting in the effective endovascular management of a fusiform aneurysm affecting both vertebral arteries (VA), following unsuccessful coil vessel sacrifice treatment.

A 61-year-old female presented with an unruptured fusiform aneurysm in the bilateral vertebral arteries (VAs). Treatment included a flow diverter in the right VA and vessel sacrifice in the left VA using Onyx-18 and coils. Despite initial success, left V4 segment recanalization occurred. Endovascular treatment, deploying two devices and additional coils using the MVP®, halted the flow. Follow-up showed left VA occlusion and reconstruction of the treated right VA, with the patient being discharged without deficits.

This case demonstrates a unique approach using MVP® alongside a flow diverter (a Pipeline^TM^ Embolization Device (PED), Medtronic) for the treatment of a V4 segment fusiform aneurysm. This innovative technique is an alternative when conventional coil embolization for vessel sacrifice fails. The MVP®'s ease of use and precise delivery render it a feasible and efficacious alternative for treating complex aneurysms.

## Introduction

Comprising less than 0.1% of all intracranial aneurysms, fusiform and dolichoectatic aneurysms of the posterior circulation are rare conditions. Fusiform and dolichoectatic aneurysms, although rare, present distinct clinical challenges compared to saccular aneurysms [1,2]. Typically found in the vertebrobasilar circulation, these aneurysms, especially when large and partially thrombosed, manifest clinical symptoms through the compression of nearby neural structures, thromboembolism, or rupture, leading to subarachnoid hemorrhage (SAH) [2,3]. Traditional microsurgical treatments involve intricate techniques, such as multiple fenestrated clipping or bypass procedures, which may pose significant difficulty.

Endoluminal reconstruction utilizing flow-diverting stents has emerged as a promising alternative [4]. Additionally, vessel sacrifice with coiling is considered, albeit carrying a risk of recurrence [5]. Recognizing the limitations of conventional embolic materials, the MVP® Micro Vascular Plug System (MVP, Minneapolis, Minnesota, USA) offers a viable solution. The MVP, originally designed for peripheral vascular occlusion, is a fully resheathable plug with controlled detachment. It has proven effective when coil or liquid embolic strategies fail to achieve complete occlusion.

This case report illustrates the successful adjunctive use of the MVP® following the deployment of a Pipeline^TM^ Embolization Device (PED) (Medtronic) for the endovascular treatment of a recanalized V4 segment fusiform aneurysm involving bilateral vertebral arteries. The patient granted informed consent for the reporting of the case, and this case report adheres to the recommendations outlined in the CAse REport (CARE) Guidelines [6].

## Case presentation

**Figure 1:**
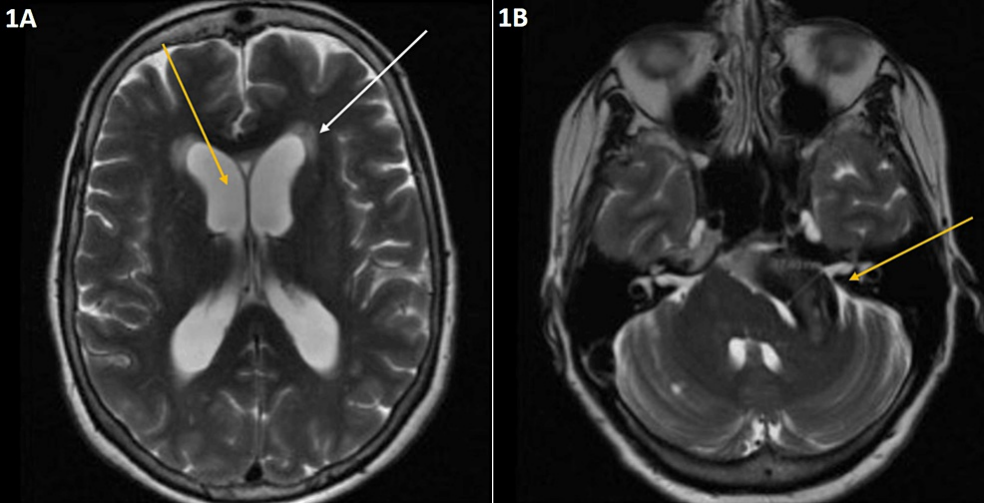
MRI T2 axial plane 1A. MRI T2 axial plane demonstrating dilated lateral ventricles (yellow arrow) with trans-ependymal transudation (white arrow). 1B. MRI T2 axial plane demonstrating a fusiform aneurysm compressing the pons (yellow arrow).

**Figure 2:**
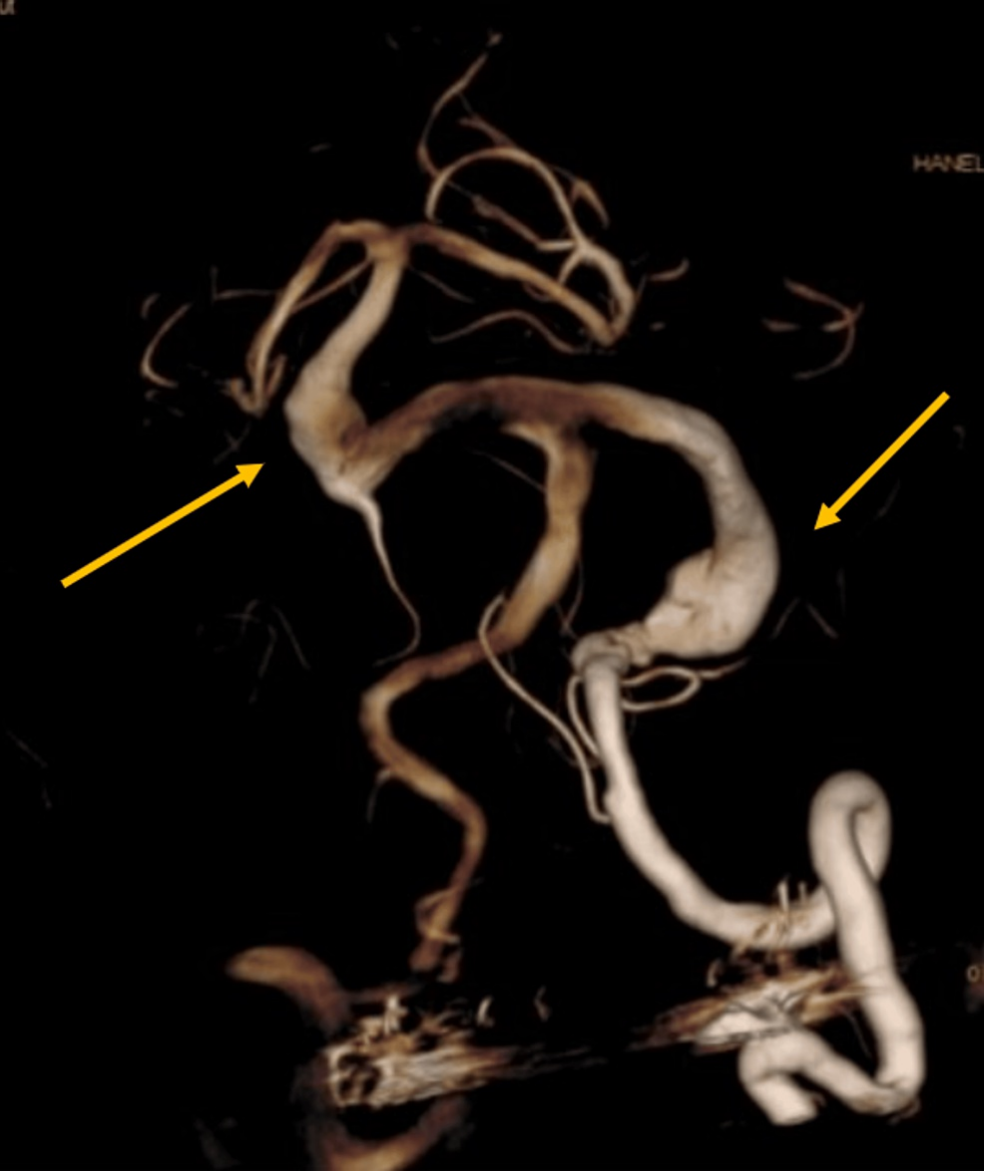
3D reconstruction angiogram demonstrating dolichoectasia of vertebrobasilar arteries

**Figure 3:**
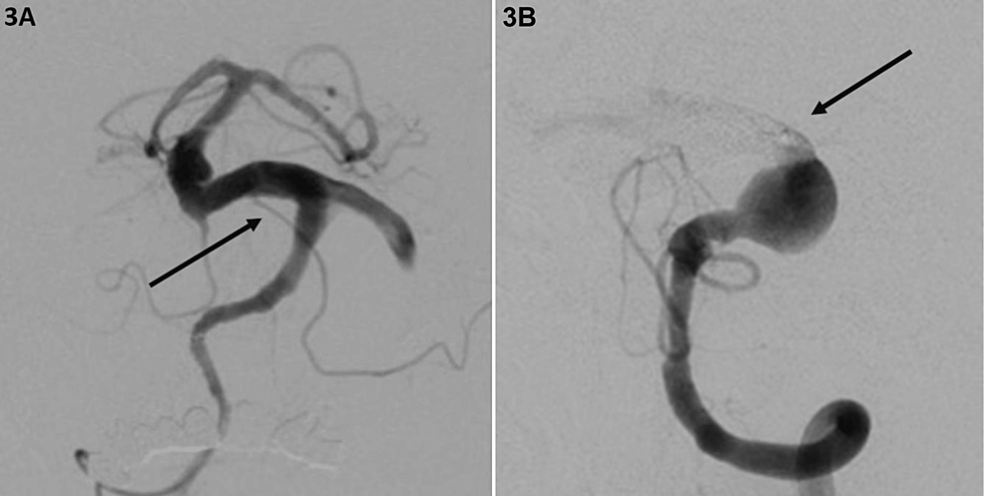
Right and left vertebral angiogram 3A. Right vertebral artery angiogram: First treatment with PED deployment. 3B. Left vertebral artery angiogram showing distal occlusion after treatment with coils and Onyx-18 (black arrow). PED: Pipeline^TM^ Embolization Device

A 61-year-old female presented with worsening diplopia, dysmetria, left-sided hearing loss, and mild right upper drift on clinical examination. MRI and MRA revealed a fusiform aneurysm involving the V4 segments of the bilateral vertebral arteries, with the left-sided aneurysm causing a mass effect on the adjacent medulla, leading to compression of the fourth ventricle and hydrocephalus (Figure 1). The patient underwent a catheter-based arteriogram, which revealed dolichoectasia affecting the proximal third of the basilar artery, along with the V4 segments of both vertebral arteries (Figure 2). Balloon-test occlusion showed no changes in the neurologic exam with occlusion of either vertebral artery. Subsequently, the patient underwent treatment with the placement of a single 4.5 x 35mm PED spanning from the right V4 segment to the basilar artery. After the PED was deployed, the left vertebral artery was sacrificed at the V4 segment using Onyx-18 (Medtronic) and Axium (Medtronic) helical coils (Figure 3).

**Figure 4:**
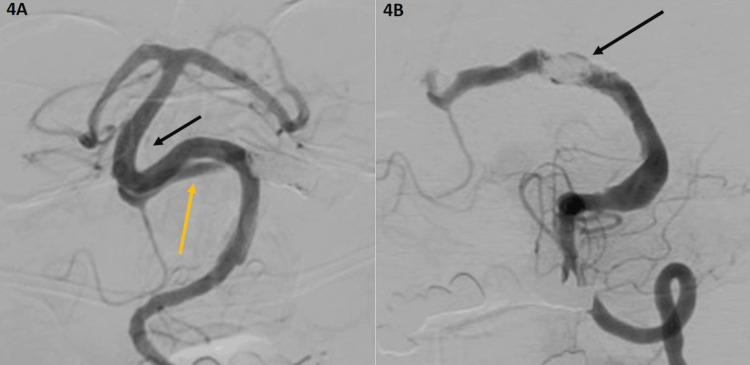
Right and left vertebral artery angiogram 4A. Right vertebral artery angiogram remodeling after three months of PED (black arrow) and retrograde flow to the left vertebral artery (yellow arrow). 4B. Left vertebral artery angiogram demonstrating recanalization of the aneurysm and flow through the coils and Onyx cast. PED: Pipeline^TM^ Embolization Device

A follow-up diagnostic angiogram performed three months later demonstrated a recanalization of the left V4 segment, with the increased size of the aneurysm (Figure 4). At this moment, the patient was asymptomatic. To halt the persistent flow into the aneurysm through the left V4 segment, the patient underwent endovascular treatment with the use of MVP for the sacrifice of the left vertebral artery. This decision was made after the patient provided written informed consent.

Vessel occlusion technique

**Figure 5:**
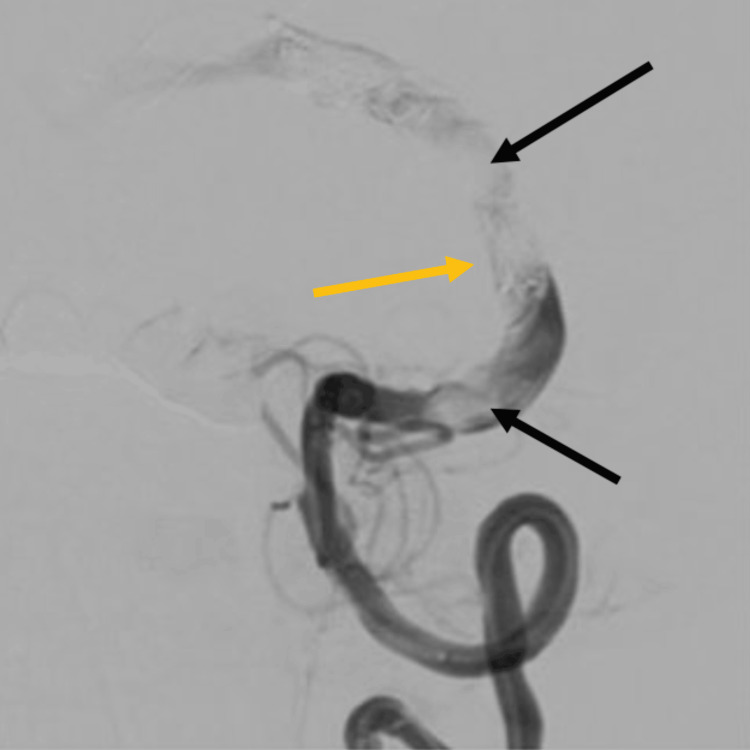
Left vertebral artery angiogram Left vertebral artery angiogram demonstrating two MVPs (black arrows) deployed after PICA origin with a second pack of coils between them (yellow arrow). MVP: Micro Vascular Plug; PICA: posterior inferior cerebellar artery

**Figure 6:**
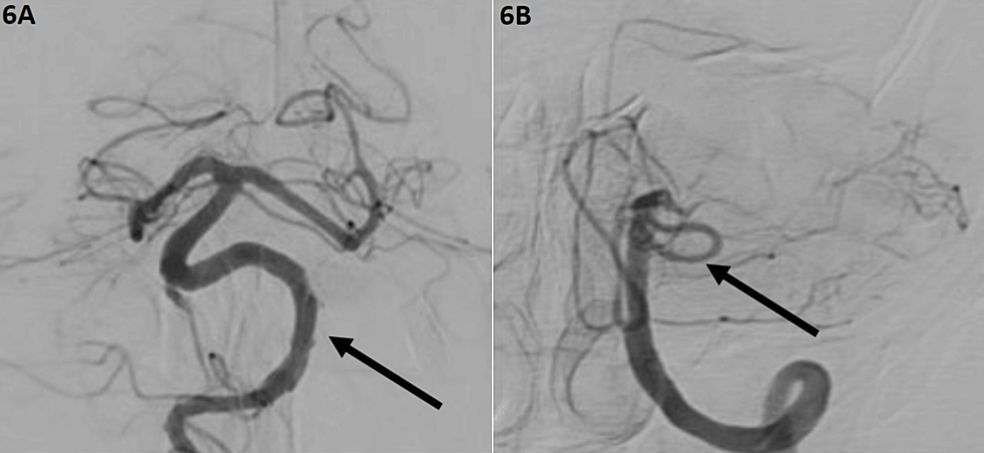
Right and left vertebral artery angiogram after treatment 6A. Right vertebral artery angiogram: final result of the right vertebral reconstruction. 6B. Left vertebral artery angiogram: final result after left vertebral artery occlusion distal to PICA. PICA: posterior inferior cerebellar artery

The right common femoral artery was used as the approach and a 6F guide catheter was positioned in the left VA. A 0.027’’ microcatheter was threaded over a 0.014’’ microwire proximal to the previously deployed coils and Onyx cast. The device size was chosen in accordance with the manufacturer's recommendations, considering that the vessel measured less than 5 mm in its largest diameter. After flushing it with heparinized saline, the MVP-5 was loaded into the microcatheter and delivered by holding the delivery wire in place and slowly retracting the microcatheter. Angiography demonstrated good positioning of the device. The device was released via anti-clockwise push-wire rotation to detach the screw under fluoroscopic guidance. Finally, the push wire was removed. A second coil cast was made, and a second MVP-5 was deployed proximal to it in the same manner. The posterior inferior cerebellar artery (PICA) was preserved. (Figure 5) The patient was discharged the next day with no neurologic deficit and without antithrombotic therapy, and follow-up angiography one month later showed total occlusion of the left VA distal to the origin of the PICA with good reconstruction of the previously treated V4 segment of the right VA (Figure 6).

## Discussion

The use of flow-diverting stents as an endovascular treatment strategy has represented a significant step forward [7,8]. Both reconstructive and deconstructive approaches demonstrate efficacy. Optimal outcomes for fusiform basilar artery aneurysms have been observed with reconstructive endovascular treatment, combined with dual antiplatelet therapy. Considering the elevated recurrence rates associated with aneurysms originating from the PICA, deconstructive strategies such as PICA occlusion warrant consideration [9].

In the case included herein, our treatment strategy was predicated on the endoluminal reconstruction of the right vertebral artery with a PED while sacrificing the left vertebral artery to reduce inflow into the aneurysm. Currently, several products are available for the endovascular occlusion of vessels, including liquid embolic agents, coils, and plug devices, each with its unique advantages and limitations. No single method of vessel occlusion is universally applicable, and some of these agents are complementary.

Liquid embolic agents, including Onyx and N-butyl cyanoacrylate, have been used for parent vessel occlusion, either by themselves or as adjuncts to other devices [10]. Despite being effective and permanent, liquid embolic agents lack the controllability of detachable devices such as coils or plugs. Their potential for distal extension or migration during vessel occlusion poses risks.

Coil embolization is a useful technique because it can create targeted and controlled vessel occlusion [11,12]. As was seen in our case, in situations in which a high flow state exists, the recanalization of the parent artery despite its sacrifice is a notable risk. Additionally, there is a risk of stroke due to the potential for thrombus formation and distal embolization [13].

We elected to utilize the MVP as an alternative after the failure of vessel sacrifice with coils and liquid embolic. The MVP is a detachable device designed for precise, targeted vessel occlusion and has been used with success in a complex, unruptured basilar/superior cerebellar artery aneurysm [14]. The MVP comes in multiple sizes: the MVP-5, with an unconstrained outer diameter of 6.5 mm, is recommended in vessels in the 3.0-5.0 mm range. The device is delivered through a 0.027” microcatheter, is resheathable, and can readily be repositioned. We deployed two MVP-5 devices, positioned proximally and distally to the aneurysm, in order to prevent its growth from both sides. This strategic placement was crucial given the reverse flow from the basilar artery into the aneurysm. Our treatment was successful after left VA sacrifice with the MVP and right VA reconstruction with the PED, finally managing the fusiform vertebral artery aneurysm. Applying this technique, the main complication that can be expected is device migration, which did not occur in this patient.

## Conclusions

The treatment of a V4 segment recanalized fusiform aneurysm affecting both vertebral arteries, demanding an unusual solution utilizing the MVP® in conjunction with the Pipeline^TM^ Embolization Device (PED), represents a novel approach leveraging the safety features of MVP for cerebral vessel occlusion. Endoluminal parent artery reconstruction using flow-diverting technology with concomitant contralateral vertebral artery sacrifice may represent an excellent treatment option for patients with posterior circulation fusiform aneurysms. The ease of use, ability to be resheathed, and precise delivery of the MVP make it a feasible, efficacious, and recommended alternative approach in the treatment of these complex aneurysms.
